# Learners’ perceptions of the medical pause: cognitive load and team performance

**DOI:** 10.3389/fpsyg.2026.1740427

**Published:** 2026-05-07

**Authors:** Joy. Y. Lee, Danielle Solish, Nicola Symonds, Michael Pierce, Heather Braund, Timothy Chaplin, Jeroen J. G. van Merrienboër, Adam Szulewski

**Affiliations:** 1 Faculty of Governance and Global Affairs, Leiden University, The Hague, Netherlands; 2 Department of Ophthalmology and Vision Sciences, University of Toronto, Toronto, ON, Canada; 3 Faculty of Health Sciences, Office of Professional Development and Educational Scholarship, Queen’s University, Kingston, ON, Canada; 4 Faculty of Health Sciences, Department of Biomedical and Molecular Sciences, Queen’s University, Kingston, ON, Canada; 5 Department of Emergency Medicine, Queen’s University, Kingston, ON, Canada; 6 School of Health Professions Education, Faculty of Health, Medicine and Life Sciences, Maastricht University, Maastricht, Netherlands; 7 Department of Psychology, Queen’s University, Kingston, ON, Canada

**Keywords:** cognitive load, leadership, medical education, mixed-methods, pause, reflection, simulation, training

## Abstract

**Introduction:**

The medical pause is a theoretical framework describing how brief interruptions in clinical activities can optimize cognitive load through reflection and relaxation. However, little is known about what occurs during instructor-prompted and learner-led pauses in simulation-based training. This study investigates whether reflection or relaxation predominates during the specific pauses and how these activities influence cognitive load and performance.

**Methods:**

Fifty-two medical students (13 teams) participated in an emergency medicine team simulation, assigned either to a pause group or a control group. A mixed-methods design was employed, combining semi-structured focus group interviews with quantitative assessments of cognitive load and performance.

**Results:**

Three themes emerged: (1) dynamic fluctuations of cognitive load at the individual level, (2) perceived usefulness of pausing, and (3) the central role of team leadership. Reflecting the dynamic nature, no significant quantitative differences were found between groups at the individual level. However, Bayesian analysis provided anecdotal evidence that leaders in the pause group experienced higher germane load, suggesting greater reflective engagement during pauses.

**Discussion:**

These findings indicate that the pauses may foster reflection, particularly among leaders. The study discusses the limitations of one-time quantitative measures in complex simulations and recommends adopting team-based rather than individual-level analyses of cognitive load and performance in future research.

## 1 Introduction

Resuscitation of critically ill patients is a complex clinical task that often imposes high cognitive demands on healthcare providers (White et al., 2018). Excessive cognitive load can contribute to preventable medical errors (Szulewski et al., 2020; Vincent et al., 2021). The *medical pause* (Lee et al., 2021) offers a theoretical framework for managing cognitive overload in such contexts. Grounded in cognitive load theory (CLT), it posits that brief interruptions in task performance allow learners to regulate cognitive load through processes such as reflection and relaxation. Yet, empirical evidence remains limited regarding what actually occurs during a learner-initiated pause. This study therefore pursues two objectives: (1) to identify the cognitive activities that take place during pauses, based on learners’ perceptions, and (2) to examine how learner-led pauses influence cognitive load and performance in simulated emergency medicine teamwork.

### 1.1 Cognitive load theory in medicine

Cognitive load theory (CLT) offers a framework for understanding human cognitive architecture as it applies to learning. CLT describes three basic elements of CL that impact working memory, the part of human cognitive architecture responsible for the temporary storage and manipulation of information during cognitive tasks (Young et al., 2014). The traditional framework divides CL into the elements of *intrinsic* CL (related to task complexity and prior knowledge), *extraneous* CL (related to suboptimal information presentation), and *germane* CL (that part of intrinsic CL used for cognitive schema construction and automation). Measured CL is thought to represent the sum of intrinsic and extraneous CL, which should not exceed working memory in an optimal learning environment (van Merriënboer and Sweller, 2010; Young et al., 2014).

CLT has been increasingly applied to performance in complex professional settings such as medicine (Szulewski et al., 2020). Simulation-based training, which recreates realistic clinical conditions through high-fidelity scenarios (Lee et al., 2024; McGaghie et al., 2010; Weller et al., 2012), exposes learners to the cognitive and emotional demands of actual resuscitation. These demands can readily exceed working-memory limits, leading to cognitive overload. Developing strategies to regulate cognitive load is therefore essential for supporting clinical performance and, ultimately, patient safety. Interventions such as cognitive aids (Lee et al., 2023) and structured stress-management training have been proposed to mitigate overload in both simulated and authentic emergency contexts (Groombridge et al., 2019).

### 1.2 The medical pause

The *medical pause* is defined as “a formal or informal decision to stop current action in medical procedures for a brief time to perform beneficial cognitive activities that enhance performance and patient safety” (Lee, 2022; Lee et al., 2021). A well-known example is the timeout, during which a clinical team temporarily suspends action to reassess the situation (Kelly et al., 2011). Originally introduced as a safety protocol to prevent procedural errors and patient harm (Lee, 2010), timeouts can be implemented formally (e.g., in pre-surgical safety checklists) or informally, as deliberate moments to “slow down when you should” in response to unexpected events or difficult situations (Moulton et al., 2010). The act of consciously stopping provides time for higher-order cognitive processes, which can enhance performance and improve quality of care.

The process of a medical pause can be described in two phases: the *decision-making phase* and *the execution phase*. During the decision-making phase, clinicians determine *when* to interrupt ongoing activity to counteract negative momentum. This requires advanced clinical judgment and situational awareness, as pausing at an inappropriate time may hinder performance (Beilock et al., 2008). Consequently, novice learners may not yet be equipped to decide when to pause (Lee et al., 2021). In team settings, decisions to initiate a pause may be supported through real-time clinical decision aids (Shear et al., 2018), or delegated to team leaders or external facilitators.

The execution phase focuses on *what* to do during the initiated pause, involving two primary cognitive activities: relaxation (i.e., cognitive rest) and reflection (i.e., reflective reorganization). Relaxation reduces cognitive overload by alleviating the sources of stress or allowing temporary cognitive rest (Parnabas et al., 2014). Such processes have been shown to decrease anxiety and support performance and learning (Lee et al., 2020; Yadav and Mutha, 2016). Reflection, in contrast, engages positive cognitive load (e.g., germane load) by reallocating cognitive resources toward schema construction and performance regulation. Through reflective processing, learners integrate working-memory and long-term-memory representations, review prior actions, and reorganize strategies to generate adaptive solutions (Lee et al., 2023,^2024^).

While the concept of the medical pause has been theoretically articulated, empirical research on its implementation in medical education remains scarce. Incorporating structured pauses into simulation-based training may help learners cultivate the ability to interrupt negative momentum and regulate cognitive load (Moulton et al., 2010; Lee et al., 2021). However, little empirical evidence exists on what cognitive activities actually occur during the execution phase of a learner-led pause in simulation contexts.

### 1.3 The present study

This study explores which cognitive processes (e.g., reflection or relaxation) predominate during a pause based on students’ perceptions. Drawing on the theoretical framework of the medical pause, we define a specific pause type: instructor-prompted and student-led pause. Because the decision of when to pause requires advanced clinical judgment that novice learners typically lack, instructors determined the timing of pauses in the present setup (i.e., instructor-prompted). However, students were given autonomy to lead the activities during each pause, allowing observation of naturally occurring cognitive processes (i.e., student-led). Using this definition, this study explores how students perceive the utility of the pauses with respect to cognitive load and team performance (RQ1). In parallel, it investigates how the pauses influence different types of cognitive load and performance outcomes (RQ2), resulting in the following two research questions:

*RQ1:* What are medical students’ perceptions of the pause taken during a simulated resuscitation scenario, in relation to cognitive load, perceived utility, and effects on performance?

*RQ2:* Given the cognitive activities occurring during the pause, how does the pause influence different types of cognitive load and performance outcomes?

## 2 Methods

### 2.1 Participants

Fifty-two medical students in their second third year participated in this study. All second- and third-year medical students from Queen’s University who had previously participated in the Competent Acute Resuscitation Through Successive Simulation (CARTSS) training program were eligible to participate. CARTSS is a medical student-led program at Queen’s University that teaches first year medical students the basic approach to cardiac arrest resuscitation (e.g., chest compressions, basic airway management, and defibrillation) in a peer-mentorship setting. Any medical student who had not completed the CARTSS program or those in first and fourth year of medical school were not eligible to participate to ensure all participants had standardized baseline experience with cardiac resuscitation training. However, their hands-on experience could vary due to COVID-19 related online training. One hundred fifty eligible medical students were contacted for recruitment in the study via email. Participation was voluntary, and no incentives or forms of compensation were provided for participants. No dropouts occurred within the participant pool. Informed written consent was obtained by all participants prior to participation.

### 2.2 Study design

A convergent mixed-methods design with an interactive approach (Fetters et al., 2013) was adopted to address the two research questions. This design was selected over a sequential approach because there was no opportunity to collect the data from the two strands (i.e., quantitative and qualitative) in a phased approach. For RQ1, semi-structured focus group interviews were conducted to explore learners’ perceptions of cognitive processes during pauses. Because of the complexity of emergency medical simulations, direct measurement of real-time processes is not feasible. A qualitative approach was therefore considered appropriate to capture learners’ subjective experiences and interpretations.

For RQ2, a quantitative comparison was performed to examine the effects of learner-led pauses on cognitive load and team performance. Participants were assigned to teams of four and randomly allocated to either the pause or control group using the RAND function in Excel. Within each team, one member was randomly designated as the team leader.

### 2.3 Procedure

All participants watched two training videos (2–3 min each) prior to beginning the simulation. Both groups were first shown the same video reviewing basic roles in simulation (cart, bag-valve mask ventilation, and chest compressions). Participants in the pause group were subsequently shown a video introducing the concept of the pause and its potential use in simulation. In contrast, participants in the control group were shown a sham video, which detailed the principles of simulation training for an equivalent duration of time. A basic ventricular fibrillation cardiac arrest simulation case lasting approximately 5 min was run for both groups. The SimMan 3G (Laerdal, Toronto, Canada) manikin was used for all scenarios. In the control group, the scenario was run without interruption. In the pause group, a 30 s pause was introduced during the scenario. Participants were encouraged to pretend as though time was frozen during the pause.

Immediately following the simulation, all participants were asked to complete a questionnaire developed by Leppink to assess CL (Leppink et al., 2014). After completion of the questionnaire, a semi-structured focus group led by one of the researchers (NS, DS, MP) was conducted with each group to debrief participants’ experiences and reflections during the simulation case. All data were collected in the simulation lab at Queen’s University School of Medicine. Ethics approval was obtained through the university’s research ethics board (file 6,036,083). The Consolidated Criteria for Reporting Qualitative Research checklist (COREQ) was used to ensure methodological rigor in the reporting of qualitative data (Tong et al., 2007).

### 2.4 The pause intervention group

A pause was initiated at a point in the scenario where CL was theorized to be high. In consultation with experts in medical education, simulation, and cognitive load theory, it was determined the pause should be initiated 10 s after a participant identified deterioration in the manikin’s condition and for the pause to last 30 s in duration. Identification of the manikin’s deterioration was indicated by one of the participants verbalizing that the manikin was not responding, stating they no longer felt a pulse, or announcing a change in the cardiac rhythm on the monitor. The pause was instructor-prompted; one of the researchers (NS, DS, MP) verbally initiated (“please pause the simulation”) and terminated the pause (“please resume the simulation”) over the intercom. After the pause was completed, the group was instructed to resume the case where they had previously left off.

The pause itself was designed to be student-led; there were no explicit instructions on what to do during the pause and participants were free to use this time however they felt was most useful at the time of the pause. The decision not to assign explicit tasks during the pause was intentional, as this study was exploratory in nature. We believed observing the spontaneously occurring activities during the pause would yield valuable insights. While participants were not assigned any specific tasks during the pause, examples were provided on how the pause could be utilized in the pre-simulation video introducing the medical pause. This included allowing the leader to summarize the scenario, reviewing next steps, or encouraging individual silent reflection. Researchers noted real-time observations during these pauses by documenting field notes during the pause. This included documentation of which team members spoke and a description of activities that took place during the pause.

### 2.5 Data collection

#### 2.5.1 Focus group interview

Participant perceptions of the pause were assessed at the end of the simulation. Specifically, after completion of the CL questionnaire, a semi-structured focus group was led by one of the researchers (NS, DS, MP) to debrief the scenario and assess overall participant experience of the simulation. Focus groups were approximately 10-min in length. These researchers received training on how to conduct and facilitate focus groups from the senior qualitative researcher on the project (HB).

#### 2.5.2 Cognitive load measure

All participants completed the 13-question Leppink CL questionnaire (Appendix 1S) after completion of the scenario to measure intrinsic, extraneous, and germane CL (Leppink et al., 2014). Participants responded on a Likert scale from 0 to 10 with 0 corresponding to “not at all” and 10 corresponding to “completely the case.” Leaders’ CL was collected at the same as all participants and compared separately to non-leaders in both pause and control groups.

#### 2.5.3 Performance measure

Performance during the simulation was evaluated using a two-section performance tool. This tool was chosen as it is used in the CARTSS programm (O’Leary et al., 2018) and has some preliminary validity evidence for the use of this tool in this context has been previously generated. The first section of the tool was graded objectively with simple “yes/no” statements based on whether the participants completed four critical checkpoints of the simulation case. The second section consisted of additional relevant quantitative outcomes measured within the simulation lab. This included time to the initiation of chest compressions from loss of pulse (in seconds), maximum compression depth (in millimetres), whether adequate compression depth (50-60 mm) was reached, and compression rate (compressions per minute). These metrics capture the initial priority actions in a ventricular fibrillation case as suggested by American Heart Association (Wigginton et al., 2025).

The relationship between simulation performance measures and cognitive load is well established in literature (Naismith and Cavalcanti, 2015; van Merriënboer and Sweller, 2010): When cognitive demands exceed a learner’s working memory capacity, the resulting cognitive overload impairs the execution of complex motor tasks. In resuscitation training, complex skills involve physical actions, patient management decision-making, and time-sensitive priorities (Szulewski et al., 2020). Empirical evidence supports the relationship between cognitive load and performance measures (e.g., task duration, the precision of resuscitation motor skills) within medical education contexts (Haji et al., 2015; Lee et al., 2019).

### 2.6 Qualitative data analysis

Semi-structured focus groups with all participants were audio-recorded and transcribed verbatim. Reflexive journaling was used throughout the data collection and analysis process. More specifically, researchers made notes of key patterns, questions, and potential assumptions as they emerged. These notes were discussed during the analysis process as part of the ongoing approach to reflexivity and formed an initial foundation for open coding followed by a more focused coding approach. This was particularly important given that some members of the research team knew the participants. This was one purposeful reason to involve HB with extensive expertise in qualitative research and someone who was external to the MD program. Thus, she had no knowledge of the participants. Focus group transcripts were independently coded inductively using NVivo software (Version 12) by three researchers (NS, DS, MP) with support from HB. One focus group transcript from each participant cohort (10% of the overall data) was selected for intercoder reliability and generation of the preliminary codebook. Longer, more complex transcripts were preferentially used at this stage to maximize code generation potential. Preliminary coding was performed by four researchers (NS, DS, MP, HB) where three focus group transcripts were coded independently by all four researchers and then compared line by line. After discussion, intercoder reliability was over 90%. This means that the coders agreed on the line-by-line coding for more than 90% of the coded segments. During the few instances where the coders disagreed, they discussed the codes until they reached consensus. This process resulted in a consensus-built codebook that was used for the remainder of the coding. This process aligns with published guidelines for conducting qualitative intercoder reliability (Cofie et al., 2022).

After the intercoder reliability check, the remaining 11 transcripts were analyzed between three researchers (NS, DS, MP). Inductive thematic analysis was subsequently conducted to explore themes (Braun and Clarke, 2006). All new codes and subthemes were discussed and critically reviewed with HB. Subthemes were grouped into overarching themes through ongoing discussion. Consensus on themes was achieved between all three researchers with regular input from HB. All members of the research team met to discuss the final themes and ensure representation across the dataset.

### 2.7 Quantitative data analysis

CL data using responses from the Leppink questionnaire were collected for quantitative analysis. The Leppink questionnaire was chosen due to its robust series of high-quality studies during derivation and validation, and its ability to differentiate between the subcomponents of cognitive load. Additionally, there is a growing body of validity evidence to support the use of this tool in simulated educational settings (Andersen and Makransky, 2021; Tremblay et al., 2017). From the questionnaire, a quantitative score based on the sum of Likert responses was calculated for each participant’s intrinsic cognitive load (ICL), extraneous cognitive load (ECL), and germane cognitive load (GCL). Performance data including compression depth, rate, and time to compressions was collected from mannikin and associated software. SPSS software (Version 28.0) was used to generate descriptive statistics between control and pause groups. Normality of the data distributions was assessed using the Shapiro–Wilk test. ICL and GCL were found to be normal. Given that the distribution of ECL was nearly normal, we proceeded with independent sample t-tests to compare the means between groups for each measured variable with post-hoc Mann–Whitney tests to confirm results. *p*-values were considered significant at 0.05.

### 2.8 Research team and reflexivity

Our reflexivity processes followed published guidelines (Braund et al., 2024) for considerations when writing reflexivity statements and the need for ongoing reflexivity throughout the research process. The research team consisted of two emergency medicine (EM) physicians with interest and training in medical education, simulation, and CL theory (AS, TC), three medical students (NS, DS, MP), one health education researcher with expertise in simulation and mixed-methods research (HB), and two external researchers with expertise in CL theory and the concept of the medical pause (JL, JvM). NS, DS and MP conducted the coding and thematic analysis in collaboration with HB. The majority of participants were personally known to NS, DS, and MP. None of the participants were personally known to HB which helped to mitigate bias. Throughout the analysis, NS, DS, MP, and HB discussed the process of data collection and reflected on any biases that could possibly emerge on an ongoing basis. When conducting the focus group interviews, special attention was paid to how the questions were asked in order to minimize bias and a focus group protocol was used to ensure consistency between groups and interviewers. Interpretation of the data and discussion of the qualitative themes were also reflected upon continuously and shared among the research team to ensure accuracy in the data.

## 3 Results

### 3.1 Perceptions on cognitive load, the pause, and performance

Three major themes were identified from the thematic analysis of the focus groups and are described in Table 1.

**Table 1:** Themes and subthemes.

Theme	1. Moments of highest cognitive load	2. Perception of pause	3. Perceived performance
Subtheme	1a: Adapting to new role 1b: History-taking 1c: Distractors 1d: Loss of pulse	2a: Focus 2b: Pause characteristics 2c: Perceived utility	3a: Challenges with simulation 3b: Positive impression of simulation 3c: Team functioning

#### 3.1.1 Theme 1: moments of highest cognitive load

All participants were asked at what point during the resuscitation scenario they felt their CL was the highest. The term CL was introduced both before and during the interview, however, other terms such as *“difficult”* and *“stressful”* were taken by the interviewer to mean the same as higher cognitive load. Regardless of their responsibility in the simulation case, it was noted that for many participants, CL increased when starting a new role or switching roles. A control group participant stated it felt overwhelming when their *“mindset was focused on switching roles and keeping track of what to do rather than the case at hand.”*

Participants in the leader role specifically identified their CL was the highest when they first entered the resuscitation scenario and had to take a history. A leader in the pause group specified *“I was expecting the patient to code immediately so it came as a surprise and so I think that the actual code aspect felt like a lot less load for me than the beginning.”* External factors were also commonly reported to increase CL. This included highly cited codes such as *equipment difficulties* and *keeping track of compressions*. Participants reported encountering these difficulties during the simulation caused them to become more distracted from the task at hand. For example, one participant remarked they *“did not know what to do with the cart”* causing them to feel more flustered or they were *“trying to learn how to use it on the spot.”*

Finally, the majority of participants stated their CL was highest when the manikin lost a pulse. One participant remarked “*it took a little bit of time because we all sort of froze for a second.”* This suggests there may have been a change in CL that participants experienced during clinical deterioration.

Learning about these moments of self-reported highest CL may inform optimal moments to incorporate pauses within simulation scenarios.

#### 3.1.2 Theme 2: perception of the pause

Given the student-led nature of the pause, there were varying uses of the pause and variable perceptions about its utility. Many participants mentioned using the pause as an opportunity to discuss next steps, allowing for re-grouping, and establishing roles. One participant specified they used the pause to *“reassess the patient, and then discuss what we are going to do going forwards”* and *“to help mentally rest.”* Real-time observation by the researchers using field-notes found that none of the groups used the pause time for silent reflection; instead, the person doing most of the speaking during the pause was the leader in all cases. Further, participants most frequently used the pause to review or assign roles, clarify outstanding questions, and discuss next priorities.

Participants also commented on the length and timing of the pause. Few participants felt the length of the pause was too short. For example, *“there was not really enough time to figure out features such as if we needed to check the pulse, or re-breathing.”* None of the participants reported they felt the pause was too long. In terms of timing, opinions were mixed about whether the pause came at an appropriate time to decrease CL or whether it should have taken place later in the scenario. For participants who found the pause helpful, many noted it helped to focus their attention and *“get us out of tunnel vision.”* For example, *“As someone who was feeling more flustered at the start, I definitely felt less flustered after the pause. I was like ‘okay I can have a second, I can gather my thoughts, I do not need to do anything right now.’”*

Many team leaders found the pause useful to help focus the attention back to the resuscitation case. One leader noted *“I feel like the pause really helped me because I think we were all a little bit flustered going into the room because it did not start off exactly the way we thought it would. So having the pause allowed us to take our hands off and regroup and actually figure out what we needed to do at that moment.”* This quote demonstrates the pause not only provided time to regroup, but consequently time for reflection-in-action as a part of the learning process.

Conversely, a minority of participants, did not perceive the pause to be helpful. These participants reported lower perceived CL in the scenario and as such felt there was little utility for them to pause. For example, *“I knew exactly what I was going to do when he started coding, and so the pause just kind of confirmed it. But I do not think [the pause] changed my cognitive load. I was looking for the bag when we walked in the room because I knew that would be my job.”* Ultimately, there was a mixed response to the perception of the pause.

#### 3.1.3 Theme 3: perceived performance

Perceived performance refers to participants’ general impressions of the simulation case. This theme was broken down into three subthemes: challenges with simulation, positive impression of the simulation, and team functioning.

Participants initially discussed overall challenges they had with the simulation case. Many participants emphasized the impact of prior knowledge and experience which influenced their comfort and perceived performance in the simulation. For example, one participant noted they felt the simulation did not go well as they “*did not know where to attach all the leads and was not sure how to use the equipment.”* Other participants believed their simulation case overall went well, with some codes including *good quality CPR was performed*, *pre-assigning roles was useful* and *rapid start to compressions*. One participant stated, *“we noticed the patient was unconscious and was not responsive and so we jumped in right away with compressions. The leader gave directions on what to do, so it felt like we followed the protocol in a standard way.”* In this case, the participant not only determined good quality CPR required quick initiation of compression, but also emphasized the importance of the leader establishing direction and guiding the resuscitation to minimize errors or delays.

The final sub-theme identified was team functioning, whereby participants discussed how their group interacted during the case and how this impacted the outcomes of the simulation. For example, “*I think everyone was on the same page, and everyone worked together really well, was supportive of each other and communicated well.”* The large role the leader plays within team functioning was also frequently discussed. For example, “*I was grateful for [the leader] at the beginning, reminding me to stop [compressions] at 30, because I forgot to count how many chest compressions I was doing…that was helpful.”* Many groups also emphasized that the leader helped the simulation run smoothly by delegating tasks, highlighting the increased responsibility and role burden placed upon the leader during the simulation case.

### 3.2 Pause effects on cognitive load and performance

There were no significant differences in baseline demographic characteristics between the pause and control groups. There were no significant differences between control group and pause groups in mean ICL, ECL, or GCL (Table 2).

**Table 2:** Descriptive statistics with *t*-test comparisons of cognitive load for control vs. pause group.

CL	Group	*n*	Mean	SD	*t*	*p*
Intrinsic	Control	24	14.4	5.4	−0.04	0.972
	Pause	28	14.5	4.4		
Extraneous	Control	24	5.8	4.7	−0.22	0.825
	Pause	28	6.1	5.6		
Germane	Control	24	28.0	6.6	−0.35	0.729
	Pause	28	28.6	6.8		

To see whether prior resuscitation experience influenced their cognitive load, we compared participants who reported prior experience to those who did not. There were no statistically significant differences in CL. However, a non-significant trend of lower CL scores was found for those with prior resuscitation experience (Table 3).

**Table 3:** Descriptivs statistics with *t*-test comparisons of cognitive load based on prior resuscitation experience.

CL	Prior experience	*n*	Mean	SD	*t*	*p*
Intrinsic	No	43	14.6	5.0	0.53	0.600
	Yes	9	13.7	4.1		
Extraneous	No	43	6.4	5.2	1.46	0.150
	Yes	9	3.7	4.3		
Germane	No	43	28.5	7.1	0.37	0.712
	Yes	9	27.6	4.3		

There were no statistically significant differences between the pause and control groups for any performance metric collected, including time to compressions (s), average compression depth (mm), and average compression rate (Table 4).

**Table 4:** performance data for pause group vs. control group.

Performance measure	Group	Mean	SD	*t*	*p*
Calculated time to compressions (s)	Control	24.0	10.5	−0.70	0.498
	Pause	28.6	12.6		
Average compression depth (mm)	Control	43.3	8.6	1.10	0.296
	Pause	39.4	3.6		
Average compression rate (compressions/min)	Control	112.5	4.5	0.85	0.413
	Pause	108.9	9.6		

### 3.3 Additional exploration: pause effects on cognitive load of leaders

Since the qualitative analysis indicated that the leadership role was central to team performance, we further examined the cognitive load of leaders only, following interactive approach of the convergent mixed-method design (Fetters et al., 2013). As reflection rather than relaxation was identified as the predominant cognitive activity among leaders, we hypothesized that leaders would experience higher cognitive load in the student-led pause condition than in the control condition.

To evaluate whether this hypothesis warrants further investigation, we conducted Bayesian independent-samples *t* test using JZS priors (Aczel et al., 2018; Rouder et al., 2009). Bayes factors in favor of the alternative hypothesis (BF_10_) was calculated to quantify how much more likely the observed data were under the alternative hypothesis than under the null. The prior distribution followed the default Cauchy with location 0 and scale of 1/√2 (Ly et al., 2016). Since our alternative hypothesis is one-sided, we truncated this distribution at zero. Analyses were performed in R (version 4.1.1; R Core Team, 2024) using the BayesFactor package (Morey and Rouder, 2018). Table 5 demonstrates the Bayes factors for each type of cognitive load for leaders. Following the categories of Jeffreys (1998), our data demonstrated weak evidence supporting the alternative hypothesis (BF_10_ = 1.67) for germane load only.

**Table 5:** Descriptive statistics with Bayes factors of cognitive load for leaders.

CL	Group	*n*	Mean	SD	BF_10_
Intrinsic	Control	6	15.7	5.69	0.47
	Pause	7	15.9	3.72	
Extraneous	Control	6	7.38	5.13	0.64
	Pause	7	9.14	7.31	
Germane	Control	6	26.5	3.71	1.67
	Pause	7	30.3	4.96	

### 3.4 Integration of qualitative and quantitative results

To integrate the qualitative and quantitative findings, a side-by-side joint display was developed (Table 6). This display aligns learners’ perceptions of cognitive load and pausing with measured cognitive load and performance outcomes, enabling comparison across data strands. Overall, the joint display highlights conceptual convergence regarding the dynamic nature of cognitive load, complementarity in explaining null quantitative findings, and role-specific effects, particularly for team leaders.

**Table 6:** Joint display integrating qualitative and quantitative findings.

Cognitive load–relevant construct	Quantitative findings	Qualitative findings	Integrated interpretation (meta-inference)
Moments of highest cognitive load	No time-specific or role-specific differences detected using post-simulation measures; no group differences in ICL, ECL, or GCL.	Participants described peaks during role transitions, leadership entry, equipment difficulties, and loss of pulse.	Conceptual convergence but methodological divergence: qualitative data reveal dynamic fluctuations not captured by one-time self-report measures.
Nature of cognitive activity during the pause	No significant group differences in intrinsic, extraneous, or germane load between pause and control groups.	Pause used for active discussion, role clarification, and planning; leaders dominated verbal activity.	Complementary findings explain null quantitative effects: pauses were cognitively active rather than restorative.
Extraneous cognitive load sources	Mean extraneous load similar between groups.	Equipment unfamiliarity, task switching, and simulator logistics described as distracting.	Divergence in sensitivity: extraneous load salient qualitatively but diluted in aggregate quantitative scores.
Leadership and germane cognitive load	Exploratory Bayesian analysis showed weak evidence of higher germane load among leaders in the pause group (BF10 = 1.67).	Leaders used pauses for regrouping, refocusing, and strategic reflection.	Strong convergence: increased germane load aligns with reflection-in-action among leaders.
Performance and team functioning	No significant differences in performance metrics between groups.	Perceived performance linked to leadership, communication, and prior experience.	Convergence across strands: leadership and team dynamics outweighed pause effects on performance.

## 4 Discussion

This study explored learners’ perceptions of the cognitive activities that occur during an Intructor-prompted and student-led pause in emergency team simulation training. Students reported a range of cognitive experiences related to three key themes: cognitive load, perceived utility of the pause, and team performance. Quantitative analyses revealed no statistically significant effects of student-led pauses on overall cognitive load or performance. However, exploratory Bayesian results suggested that team leaders may experience increased germane load, opening up a possibility of engagement in reflective processes during pauses.

The findings for RQ1 indicate that cognitive activities during a student-led pause are heterogeneous rather than uniform. The qualitative analysis identified variations in how students perceived and utilized the pause. While some viewed it as an opportunity to reassess priorities, clarify next steps, or redistribute team roles, others reported limited benefit due to time constraints or low perceived cognitive demand. A few participants noted that their cognitive load remained consistently manageable throughout the simulation. These findings support the variability of pausing as a cognitive strategy in high-pressure team environments. Within the theoretical framework of the medical pause, both relaxation (i.e., cognitive rest) and reflection (i.e., reflective reorganization) appeared to be identified.

We consider that this diversity may stem from two primary factors: (1) variation in team roles inherent to collective performance, and (2) differences in learners’ prior knowledge. In resuscitation simulations, roles (e.g., team leader, circulation manager, timekeeper, and support member) involve distinct cognitive demands. Our qualitative data suggest that some non-leader participants did not experience high cognitive load, possibly because their tasks were narrower in scope, less complex, or externally directed by the leader. These participants may have been less affected by the pause itself, perceiving it as the leader’s responsibility to guide the team’s actions during that time.

Differences in prior knowledge likely also contributed to the variability in perceived cognitive load. Prior research has shown that learners’ existing knowledge influences both cognitive load and performance (Lee et al., 2019). In our study, prior resuscitation experience appeared to shape how participants managed cognitive demands during the simulation. Although all participants were medical students at comparable stages of training, disparities could arise from different learning experiences during the COVID-19 pandemic. Some had completed resuscitation training online only, whereas others had physical in-person practice in the simulation lab. For those without prior exposure, the environment and task demands were novel, potentially increasing cognitive load. By contrast, students with prior lab experience appeared to engage more comfortably with the scenario.

The findings for RQ2 indicated that the pause did not exert a consistent effect across participants. Three possible explanations can be considered: (1) variability in cognitive activities during the pause, (2) limited self-regulation among students, and (3) measurement constraints. First, as reflected in the results of RQ1, the diversity of cognitive activities likely contributed to the absence of a clear directional effect. Differences in team roles and prior knowledge may have led some learners to engage in reflective activities that increased germane load, while others used the pause for relaxation or remained cognitively passive. As a result, individual cognitive load trajectories diverged, masking any aggregate effects on overall load or performance.

Second, the lack of significant group differences may stem from insufficient self-regulation. Because students were instructed to self-direct the activities during the pause, many may not have known how to use the time effectively for either reflection or relaxation. Learners in the pre-clinical phase might be still developing the metacognitive and regulatory skills necessary to structure their own learning. In this context, unguided pauses may have introduced uncertainty or divided attention, reducing their potential benefit. Providing explicit guidance on how to utilize the pause might have led to more consistent outcomes.

Third, limitations in measurement may have contributed to the null findings. Cognitive load was assessed using the Leppink scale (Leppink et al., 2014), a one-time self-report measure administered after the task. Such post-hoc ratings cannot capture real-time fluctuations in cognitive load that occur during dynamic simulations. Physiological measures (e.g., galvanic skin response, pupillometry) (Ayres et al., 2021) offer higher temporal sensitivity and could complement psychometric instruments (Szulewski et al., 2017). However, the feasibility of these methods was limited in the current high-fidelity resuscitation setting. Future research should integrate multimodal approaches to capture the temporal dynamics of cognitive load during simulation-based team performance.

The exploratory analysis provided anecdotal evidence of higher cognitive load among leaders in the pause condition, suggesting increased engagement in reflective processes compared to the control condition. While this finding does not fully explain the broader effects of the pause, it underscores the central role of leadership in team-based resuscitation. Altpeter et al. (2007) similarly observed that effective surgeon leadership was critical for initiating and maintaining successful surgical timeouts. In the present study, leaders were assigned to their roles immediately before the simulation, without extended preparation. Leading a team under such time pressure likely imposed additional cognitive demands. We propose that leaders’ cognitive load may serve as a more sensitive indicator of the effects of pausing in team-based contexts. Based on this hypothesis-generating finding, future studies should focus on leaders’ cognitive processes, while accounting for the smaller sample sizes inherent in subgroup analyses.

Importantly, the observed increase took place in germane cognitive load, indicating greater reflective engagement and learning among leaders. This heightened load may represent productive cognitive effort, aligning with *secondary load* (i.e., resources devoted to self-regulation) within the medical pause framework (Lee et al., 2021). These effects are further elucidated by *desirable difficulty*, which posits that learning tasks requiring greater cognitive effort in the short term facilitate stronger long-term retention and transfer (Nelson and Eliasz, 2023). Similarly, cognitive load theory suggests that intentionally increasing germane load can facilitate deeper learning and improved performance (van Merriënboer and Sweller, 2010). Future research should examine how learner-led pauses can be designed to foster such productive engagement (e.g., germane or secondary load), particularly for team leaders during simulation-based training.

### 4.1 Limitations and future directions

This study has several limitations that should be addressed in future research. First, the sample size was relatively small due to the logistical constraints of emergency team simulations. Replicating the study with larger cohorts would improve the robustness and generalizability of the findings. Second, cognitive load was assessed using a one-time self-report measure, which lacks the temporal sensitivity for the dynamic cognitive fluctuations that occur during simulation, given that the pause occurs mid-scenario. Future studies could incorporate real-time physiological measures (e.g., heart rate variability, galvanic skin response, eye-tracking indices) to provide continuous and objective indicators of cognitive load.

Third, analyses were conducted at the individual level, even though the task required coordinated team performance. Future research should employ frameworks that account for collective cognitive processes, such as team reflexivity (Schmutz et al., 2018), and examine how team-level dynamics influence cognitive load regulation. Given the distinct role of leadership, special attention should be directed toward leaders’ cognitive load, which may be more representative of team-level cognitive demands. Fourth, the timing of pauses was determined by instructors to standardize conditions. To understand the impact of decision-making autonomy, future studies should compare instructor-initiated and learner-initiated pauses to assess how self-regulated pausing influences cognitive and performance outcomes.

Fifth, as an explorative study, the present design did not control for the type of cognitive activity undertaken during the pause. As reflection and relaxation may exert different effects on cognitive load, future research should systematically vary the nature of pause activities to identify coherent patterns. Finally, differences in prior knowledge and hands-on experience due to COVID-19-related online training were not controlled for, despite their known influence on cognitive load and performance. Future studies should account for varying levels of prior training and physical simulation experience to clarify the potential confounding effects.

## 5 Conclusion

This study investigated how medical students perceive and use learner-led pauses during simulated resuscitation and how such pauses affect cognitive load and performance. The qualitative findings revealed heterogeneous cognitive activities during pauses, including reflection, reorganization, relaxation, and refocusing. This variability likely contributed to the absence of consistent quantitative differences in cognitive load or performance between the pause and control groups. Exploratory Bayesian analysis, however, indicated that leaders in the pause condition experienced higher germane cognitive load, suggesting deeper reflective engagement during pauses. This finding underscores the central role of leadership in regulating team cognition and highlights the medical pause as a potential mechanism for fostering reflective learning among leaders.

The study also illustrates the methodological challenges inherent in examining cognitive load in dynamic team settings. Mixed-method approaches (e.g., focus group interviews, self-report scales) provide valuable triangulation but remain constrained when relying solely on post-simulation measures. Future research should adopt team-level analyses, integrate real-time physiological or behavioral indicators of cognitive load, and systematically vary the structure and guidance of pauses to better understand how these factors shape learning and performance in medical simulation.

## Data Availability

The raw data supporting the conclusions of this article will be made available by the authors, without undue reservation.
